# Anisotropic Vibration Tactile Model and Human Factor Analysis for a Piezoelectric Tactile Feedback Device

**DOI:** 10.3390/mi10070448

**Published:** 2019-07-03

**Authors:** Jichun Xing, Huajun Li, Dechun Liu

**Affiliations:** School of Mechanical Engineering, Yanshan University, Qinhuangdao 066004, China

**Keywords:** ciliary bodies touch beam, piezoelectric tactile feedback devices, anisotropic vibration tactile model, human factor experiment

## Abstract

Tactile feedback technology has important development prospects in interactive technology. In order to enrich the tactile sense of haptic devices under simple control, a piezoelectric haptic feedback device is proposed. The piezoelectric tactile feedback device can realize tactile changes in different excitation voltage amplitudes, different excitation frequencies, and different directions through the ciliary body structure. The principle of the anisotropic vibration of the ciliary body structure was analyzed here, and a tactile model was established. The equivalent friction coefficient under full-coverage and local-coverage of the skin of the touch beam was deduced and solved. The effect of system parameters on the friction coefficient was analyzed. The results showed that in the full-coverage, the tactile effect is mainly affected by the proportion of the same directional ciliary bodies and the excitation frequency. The larger the proportion of the same direction ciliary body is, the smaller the coefficient of friction is. The larger the excitation frequency is, the greater the coefficient of friction is. In the local-coverage, the tactile effect is mainly affected by the touch position and voltage amplitude. When changing the touch pressure, it has a certain effect on the change of touch, but it is relatively weak. The experiment on the sliding friction of a cantilever touch beam and the experiment of human factor were conducted. The experimental results of the sliding friction experiment are basically consistent with the theoretical calculations. In the human factor experiment, the effects of haptic regulation are mainly affected by voltage or structure of the ciliary bodies.

## 1. Introduction

Tactile feedback technology reproduces the tactile sensation for the user through a force, vibration, or other excitation methods [1]. Touch might be the most complex sensing modality compared to sight, hearing, smell, and taste [2]. The technology can be applied to assist the creation and control of virtual scenes and enhance the remote control of machinery and equipment. Tactile feedback technology is usually applied to tactile displays, or touch sensors and other equipment [3]. Some devices are able to reproduce the tactile of the textured surfaces under the finger [4,5]. Tactile feedback technology has been successfully applied in virtual reality gloves, virtual medical, tactile display, and other fields [6]. According to the excitation method, the tactile feedback device can be classified into a pneumatic type, an electromagnetic type, and a piezoelectric type. Among them, the tactile feedback device using piezoelectric materials has received wide attention and application. Piezoelectric tactile feedback devices have stable vibration, slow adaptability to vibration, and low stimulation, etc. [7,8,9]. Additionally, the response time of piezoelectric materials is relatively short, which is suitable for long-term tactile simulation [10].

The first category uses an ultrasonic vibration of piezoelectric material to modulate the contact effect between a user’s fingertip and a vibrating surface [11]. For example, Ma Lu et al. designed a tactile reproduction system based on friction control, which realizes tactile shape reproduction by controlling the frequency of the finger in different positions on the tactile plane [12]. It can be explained either by the squeeze effect or by intermittent contact of the vibrating surface with the finger [13,14]. Other reports focus on tactile displays realized with stimulator arrays, in which mechanical vibrations are generated to stimulate the mechanoreceptors of the skin. For example, in 2002, Yasushi proposed a TextureExplorer that combines tactile and force stimulators to present virtual textures to the user’s fingertip. This provided a vibration pin-array excited by piezoelectric plates employed for tactile stimulation in conjunction with the PHANToM, which is a device for force reflection to perform haptic texture rendering [15]. Hayward from McGill University presented a tactile feedback device based on the principle of piezoelectric lateral skin stretching, which was constructed from an array of 64 closely packed piezoelectric actuators connected to a membrane. The deformations of this membrane cause an array of 112 skin contactors to create programmable lateral stress fields in the skin of the finger pad [16].

Tactile feedback devices based on friction control mostly use a combination of sensors and actuators. The virtual touch is realized by detecting the spatial position of the finger to generate a vibration stimulus or to electrostatically stimulate the skin. However, due to a single stimulation method, the effect of tactile stimulation is not optimistic. The tactile effect is relatively simple, and the control system is generally more complicated. The tactile feedback techniques based on stimulator arrays can perform haptic texture rendering, but the resolution is related to the number of used actuators. Therefore, the application of a large number of piezoelectric actuators leads to manufacturing difficulties and high manufacturing costs [17].

To solve the above problems and take advantage of these two technologies, here, we arrange the array tooth structure on ultrasonically vibrating piezoelectric beams, which we call the ciliary body piezoelectric beam. The beam can be any material with elastic properties. So, in this paper, a piezoelectric tactile feedback device with a ciliary body structure is proposed, which has the features of a simple structure and control system. This technique can achieve different touch sensations depending on the direction of finger movement at the same contact position [18]. Firstly, the principle of the anisotropic vibration of the ciliary body structure is analyzed, and a tactile model of the sliding vibration of the anisotropic vibration is established. Secondly, the equivalent friction coefficient of the skin and the touch beam under full-coverage and local-coverage are deduced and solved. The change law of the equivalent friction coefficient is analyzed according to the solution results. Finally, the important parameters, such as the proportion of ciliary bodies in the same direction and ciliary bodies’ density, are changed. The equivalent friction coefficient function is solved. The effect of each parameter on the friction coefficient is analyzed, and the tactile control law and scheme are obtained.

## 2. Principle 

**Figure 1:**
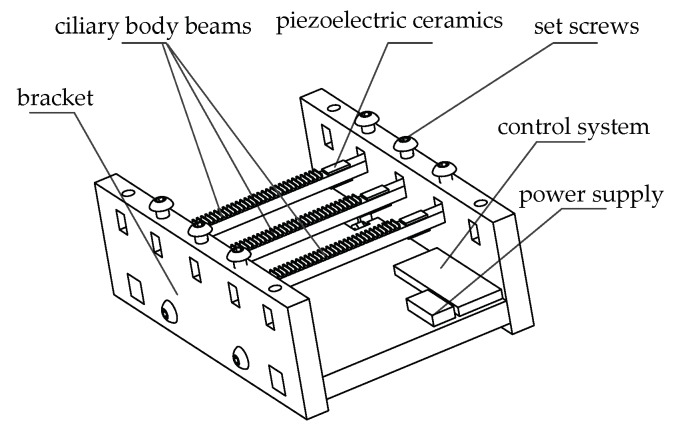
The schematic diagram of the piezoelectric tactile feedback device.

The structure of the piezoelectric tactile feedback device is shown in Figure 1, mainly including set screws, piezoelectric ceramics, ciliary body touch beams, bracket, control system, and power supply, of which the control system includes an analog-to-digital conversion module, power amplifier modules, and Bluetooth modules.

**Figure 2:**
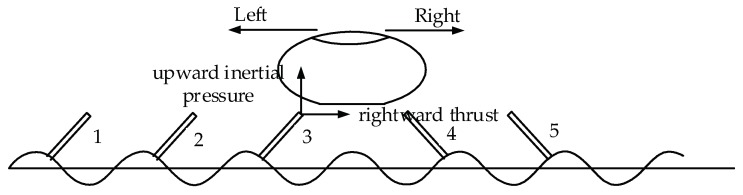
Working principle.

When a sinusoidal signal with a frequency close to the natural frequency of the touch beam is supplied to the piezoelectric sheets, the touch beam resonates and produces a bending vibration. The ciliary body at different positions of the touch beam will vibrate in different directions. As shown in Figure 2, the ciliary bodies under the finger are distributed on the right and the left side of the vibration peak. The ciliary bodies of 1, 2, and 3 indicate the ciliary body distributed on the right side of the peak, and 4 and 5 indicate the ciliary body distributed on the left side of the peak. At this time, the ciliary bodies distributed on the right side of the peak will give the finger upward inertial pressure and rightward thrust. The ciliary bodies distributed on the left side of the peak will give the finger upward inertial force and leftward thrust. Therefore, when one finger moves to the right, the vibration direction of the ciliary body on the right side of the peak is the same as the direction of movement of the finger. The vibration direction of the ciliary body on the left side of the peak is opposite to the direction of movement of the finger. When the finger touches the beam, the coefficient of friction of the different parts is constantly changing, which makes the subject feel that the ciliary body on the right side of the peak is smoother than the left side. When the finger moves to the left, the result is reversed. The change in tactile sensation is due to the fact that the equivalent friction coefficient between the finger and the plane is modulated by the vibrating ciliary body beam, and the sliding friction coefficient between the finger and the contact surface depends on the ratio of the ciliary body in both directions covered by the finger. The change in the frequency of the excitation signal causes the vibration mode of the touch beam to change. When the excitation frequency is close to the natural frequency, the acceleration increases, and decreases away from the natural frequency. When the excitation frequency approaches the next natural frequency, the vibration mode of the beam is also changed, and the distribution of the vibration direction of the ciliary bodies are changed as the changing of the vibration mode. The tactile effect is different compared to the previous vibration mode.

**Figure 3:**
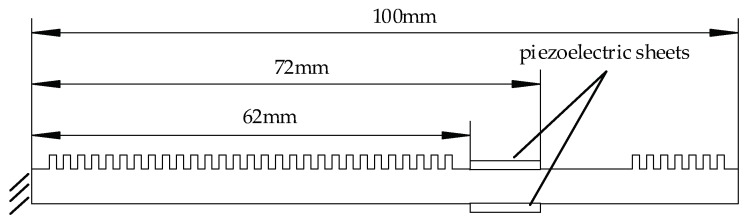
Structure of the ciliary body touch beam.

The structure of the ciliary body touch beam is shown in Figure 3. The ciliary bodies’ density and number can set multiple sets of data. It can produce a variety of tactile sensations. Piezoelectric sheets are pasted on the upper and lower surfaces of the touch beam, and the position of the paste is one peak of the mode function of the touch beam.

**Figure 4:**
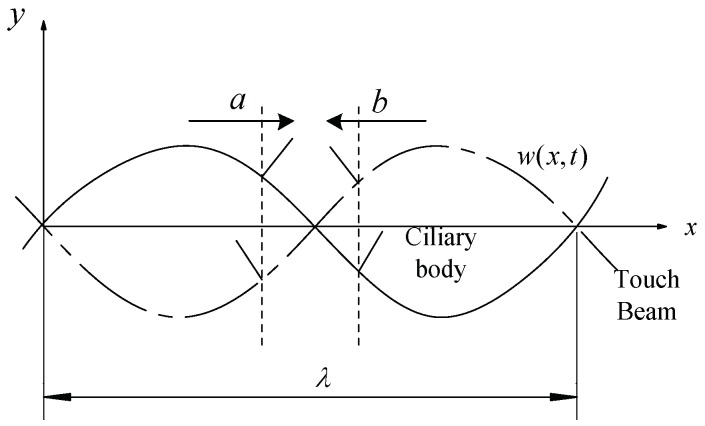
Anisotropic vibration dynamic model of ciliary bodies.

Since the ciliary body touch beam accomplishes different surface roughness by the anisotropic vibration of ciliary bodies, the number of the ciliary bodies in the two vibrating directions is the main factor in controlling the overall equivalent friction coefficient under the skin-covering length. As shown in Figure 4, the ciliary bodies’ anisotropic vibration dynamic model has a vibration of a wavelength, λ. When the ciliary touch beam vibrates between the solid line and the dashed line, the ciliary bodies will squeeze the skin from the bottom to the top. In the process of squeezing the finger skin upwards, the ciliary bodies, *a*, will move from left to right in the horizontal direction, and the ciliary bodies, *b*, will move from right to left in the horizontal direction.

Let the skin move from left to right at a certain speed on the touch beam slowly. When the skin moves above the ciliary body, *a*, the horizontal component of the vibrational direction of the ciliary body is the same as the direction of movement of the skin, and gives the skin a certain thrust along the direction of motion and reduces the total sliding friction between the skin and the touch beam. Therefore, the equivalent friction coefficient of the skin and the touch beam will be reduced by the total sliding friction of the ciliary bodies. When the skin moves above the ciliary body, *b*, the horizontal component of the vibration direction of the ciliary body is opposite to the direction of skin movement, which gives the skin the opposite directional resistance. Therefore, the equivalent friction coefficient of the skin and the touch beam affected by the total sliding friction of the ciliary bodies will increase. In the process of moving the skin, the friction coefficient decreases when the ciliary bodies with the same vibration direction as the skin movement direction are touched, and the tactility of the touch beam becomes smooth. The friction coefficient increases when the ciliary bodies with the opposite vibration direction as the skin movement direction are touched and the tactility of the touch beam becomes rough. Additionally, the vibrating direction of ciliary bodies is distributed according to the following Equation (1):

(1) $$\left\{ \begin{array}{l} \phi^{\left(i\right)}(x)\cdot \phi^{{\left(i\right)}^{\prime }}(x)<0,\;\mathrm{Right}\;\mathrm{vibration}\;\mathrm{area}\\ \phi^{\left(i\right)}(x)\cdot \phi^{{\left(i\right)}^{\prime }}(x)>0,\;\mathrm{Left}\;\mathrm{vibration}\;\mathrm{area}\; \end{array}\right.,$$

where $\phi^{\left(i\right)}(x)$ is the *i*-th order mode function of the touch beam [19].

**Figure 5:**
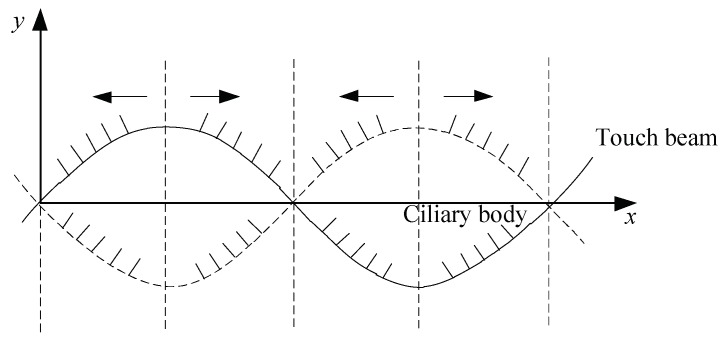
The distribution of the different directional ciliary bodies.

As shown in Figure 5, we assumed that the ciliary bodies’ density on the touch beam is sufficiently large. It can be seen that the directions of vibration of the ciliary bodies change at the peak or trough of a wave. Hence, when one finger’s skin moves from left to right, the increase and decrease of the friction coefficient shows a periodic variation pattern.

However, if the direction of the vibration of the ciliary body covered by the finger skin is positive and opposite when the finger touches the beam, the sense of touch perceived by the receptor is determined by the ratio of the two vibration directions covered by the skin. In addition, the length of the beam covered by the receptor with a single finger and multi-fingers is not the same. Additionally, the single finger covers less than half of the wavelength of the vibration form. This is called local coverage here. Multi-fingers cover the range of more than one wavelength of the vibration form. This is called full-coverage. Therefore, the tactile model under both full-coverage and local-coverage needs to be analyzed and calculated separately.

## 3. Analysis for Anisotropic Vibrating Tactile Models

### 3.1. Analysis for Full-Coverage Anisotropic Vibration Tactile Model

**Figure 6:**
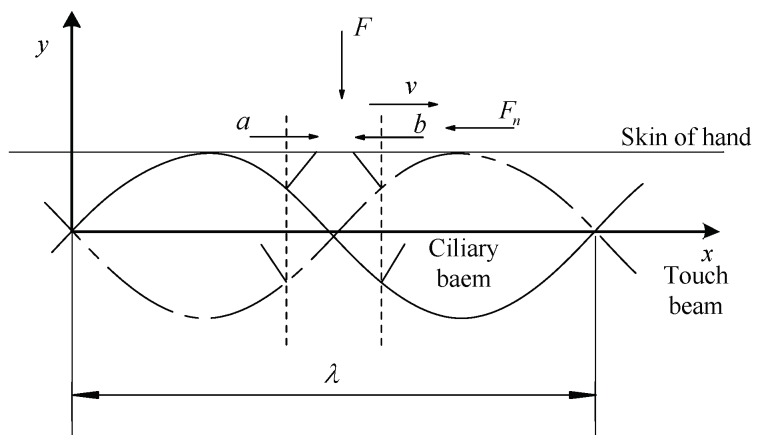
Anisotropic vibration tactile model of full-coverage.

The full-coverage anisotropic vibration tactile model is shown in Figure 6. The skin covers the full touch beam and moves to the right at a uniform velocity, *v*. The skin gives pressure to the touch beam, and the pressure between the skin and the touch beam includes the static pressure and the inertia pressure, which are given to the skin during the vibration of the ciliary bodies. Therefore, the total pressure between the skin and the touch beam can be written as:

(2) $$F=(a+b)f+f_{p},$$

where *a* is the number of vibrating ciliary bodies in the same direction, *b* is the number of vibrating ciliary bodies in the opposite direction, *f* is the static pressure of the skin on each ciliary body, and *f_p_* is the ciliary bodies’ inertial pressure on the skin of the hand.

According to the previous analysis, we know the total sliding friction includes the static pressure, the inertial pressure, and the sliding friction caused by lateral vibration of the ciliary bodies. From Equation (2), the total sliding friction force of the hand skin in full-coverage is:

(3) $$\begin{array}{l} F_{n}^{\left(f\right)}=\mu (fa+fb)+\eta (\mu f\cdot b-\mu f\cdot a)+\mu f_{p}\\ \;=\frac{3}{2}\mu fb+\frac{1}{2}\mu fa+\mu f_{p} \end{array},$$

where *μ* is the general sliding friction coefficient between the finger and the touch beam. *η* is the effective coefficient of the anisotropic vibration of the ciliary bodies. The effect coefficient of the ciliary bodies’ anisotropic vibration is the degree of the effect of the skin of the ciliary bodies in the process of touching the beam. The ciliary bodies have a more obvious effect on the skin when moving upwards. However, when the ciliary bodies move away from the skin, the effect is faint. So, here, the effect on the fingers is negligible. Therefore, the vibrating inertial force acting time of the ciliary bodies can be approximated as 1/2 of the total time, so *η* = 1/2 is taken.

Let the total number of ciliary bodies remain unchanged on the touch beam. In order to simplify the ciliary bodies’ tactile model, it was assumed that the ciliary bodies are dense enough. From Equation (3), the total sliding friction force of the hand skin in full-coverage can be written as:

(4) $$F_{n}^{\left(f\right)}=\mu (\frac{3}{2}-\frac{a}{u}+\frac{f_{p}}{uf})F.$$

Then, the equivalent friction coefficient can be written as:

(5) $$\mu^{\prime }=\mu (\frac{3}{2}-\frac{a}{u}+\frac{f_{p}}{uf}),$$

where *u* is the total number of ciliary bodies on the touch beam. *μ’* is the equivalent friction coefficient under the vibration state.

Maintaining the proportion of the same direction of ciliary bodies and the opposite direction of ciliary bodies, let:

(6) $$\frac{a}{b}=\chi ,\;\chi \in N.$$

Substituting Equation (6) into (5), we can obtain the relationship between the equivalent friction coefficient and the total number of the ciliary bodies as:

(7) $$\mu^{\prime }=\mu \left[(\frac{3}{2}-\frac{\chi }{\chi +1})+\frac{f_{p}}{uf}\right].$$

**Figure 7:**
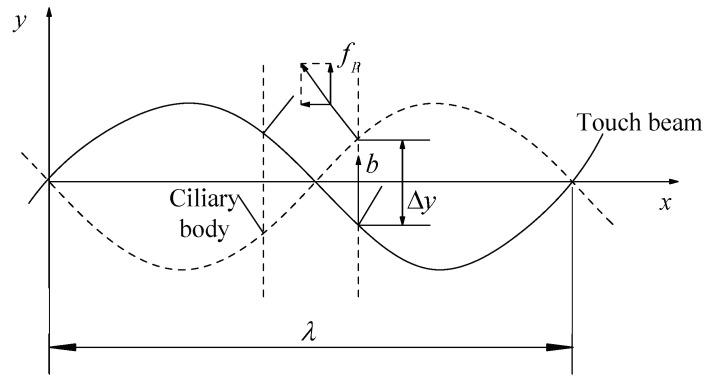
The inertial pressure of the ciliary body.

During the contact of the skin with the touch beam, the inertial pressure also affects the sliding friction between the skin and the touch beam. As shown in Figure 7, the touch beam vibrates from the solid line to the dotted line. One vibrating ciliary body produces a normal pressure on the skin that is the inertia force, *f_p_*. The inertial pressure is related to the forced response of the touch beam. The effect coefficient of the inertial pressure, *η_p_*, and the effect coefficient, *η*, of the anisotropic vibration are the same, taking *η_p_* = 1/2, so the inertial force of the ciliary bodies can be expressed as:

(8) $$f_{p}=\eta_{p}a_{c}^{\left(f\right)}m=\frac{m}{T}\cdot \sum_{j=1}^{u}\left[\int_{0}^{\frac{T}{2}}\left|w^{{\left(i\right)}^{\prime \prime }}(x_{j},t)\right|dt\right],$$

where $w^{{\left(i\right)}^{\prime \prime }}$ is the acceleration response of the touch beam, *m* is the mass of one ciliary body, and *a_c_^(f)^* is the average acceleration over one vibration period of all ciliary bodies. *x_j_* is the positional coordinate of the *j*-th ciliary body. *T* is the vibrational cycle time of the touch beam.

In the case where the density of the ciliary bodies is sufficient, one ciliary body can be approximated as a micro-element, and then the inertial pressure of the full-coverage ciliary bodies can be expressed as:

(9) $$f_{p}^{\left(f\right)}=\frac{1}{2}a_{c}m=\frac{m}{T}\cdot \int_{0}^{L}\int_{0}^{\frac{T}{2}}\left|w^{{\left(i\right)}^{\prime \prime }}(x_{j},t)\right|dtdx_{j}.$$

Substituting Equation (9) into (5), the equivalent friction coefficient in full-coverage can be shown by:

(10) $$\mu^{\prime }=\mu \left(\frac{3}{2}-\frac{a}{u}+\frac{m}{Tuf}\cdot \int_{0}^{L}\int_{0}^{\frac{T}{2}}\left|w^{{\left(i\right)}^{\prime \prime }}(x_{j},t)\right|dtdx_{j}\right).$$

The cantilever touch beam was chosen as the research object, and the relevant parameters of the piezoelectric sheet and touch beam are shown in Table 1 and Table 2. The sliding friction coefficient of the skin and the copper was *μ* = 0.4, and the parameters related to ciliary bodies are shown in Table 3.

**Figure 8:**
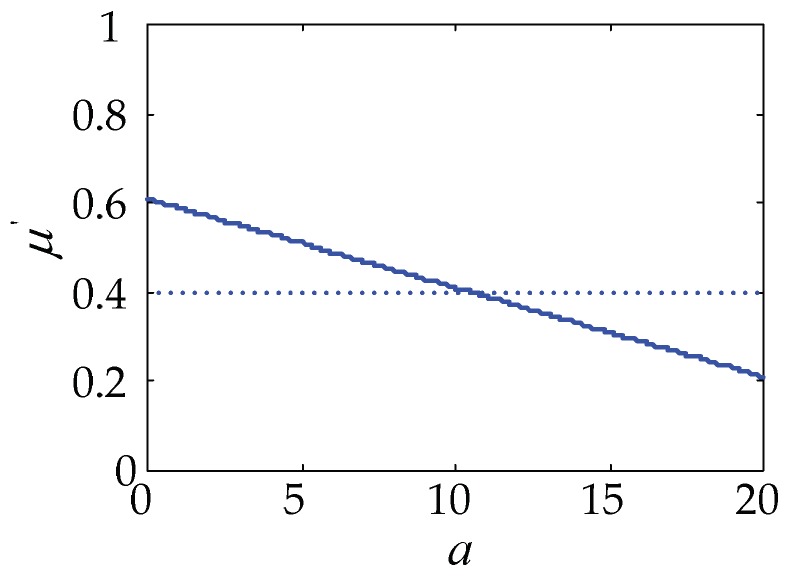
The relationship between *μ*’ and *a*.

The relationship between the equivalent friction coefficient of the cantilever touch beam and the number of the vibration direction, *a*, of the ciliary bodies in full-coverage calculated from Equation (10) is shown in Figure 8.

Figure 8 shows that if the total number of ciliary bodies on the touch beam is 20, the equivalent friction coefficient between the touch beam and the skin gradually decreases with the increase in the number of vibrating ciliary bodies in the same direction, and the two change linearly. When the number of the same directional ciliary bodies is less than half, the sliding friction force of the touch beam is greater than the general sliding friction force, and it is in the rougher state. When the number of the same directional ciliary bodies is more than half, the sliding friction force of the touch beam is smaller than the general sliding friction force and is in the smoother state. The equivalent friction coefficient of the cantilever touch beam varies from about 0.2 to 0.6.

### 3.2. Analysis for Anisotropic Vibration Tactile Model in Local-Coverage

**Figure 9:**
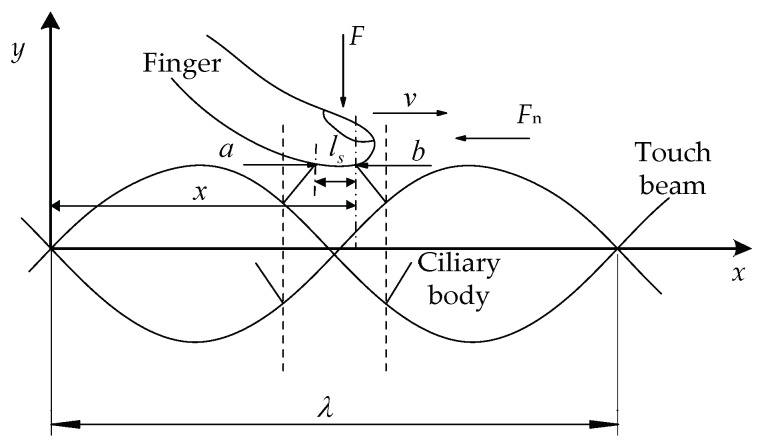
Anisotropic vibrational tactile model of local-coverage.

The local-coverage anisotropic vibration tactile model is shown in Figure 9. The skin of the finger covers the local length, *l_s_*, of the touch beam and moves to the right at a uniform velocity, *v*. The finger gives pressure to the touch beam. There are a certain number of the same directional ciliary bodies and the opposite directional ciliary bodies under the skin. The pressure between the skin of the finger and the touch beam also includes the static pressure and the inertia pressure, which is given to the finger during the vibration of the ciliary bodies. Due to the changes of the ciliary bodies’ vibration distribution state during the constant movement of the finger, the changeable law of the equivalent friction coefficient with two parts of the pressure at different positions, *x*, must be analyzed.

In the case where the ciliary bodies’ density is sufficient, every ciliary body is approximated as a micro-element. One-half vibration cycle of each ciliary body is its effective period on the finger. So, from Equation (9), the inertial pressure in the local-coverage can yield:

(11) $$f_{p}^{\left(l\right)}=\frac{1}{2}a_{c}^{\left(l\right)}m=\frac{m}{T}\cdot \int_{x_{j}-l_{s}}^{x_{j}}\int_{0}^{\frac{T}{2}}\left|w^{{\left(i\right)}^{\prime \prime }}(x_{j},t)\right|dtdx_{j},$$

where $a_{c}^{\left(l\right)}$ is the average acceleration within a half vibration period of the locally covering ciliary bodies; ls is the local-coverage length of the finger. Substituting Equation (11) to (5), the equivalent friction coefficient in local-coverage can be shown as:

(12) $$\mu^{\prime }=\mu \left(\frac{3}{2}-\frac{a}{u}+\frac{m}{Tuf}\cdot \int_{x_{j}-l_{s}}^{x_{j}}\int_{0}^{\frac{T}{2}}\left|w^{{\left(i\right)}^{\prime \prime }}(x_{j},t)\right|dtdx_{j}\right).$$

From Equation (12), it is apparent that the equivalent friction coefficient under local-coverage is mainly affected by two parts. One is the inertial force, *f_p_*, of the ciliary bodies, and the other is the proportion, *a*/*u*, of the total number of ciliary bodies in the same direction of vibrating ciliary bodies. The functional relationship between *f_p_* and the positional coordinate, *x*, was obtained from Equation (11), and the functional relationship between *a*/*u* and the positional coordinate, *x*, needs to be further analyzed.

**Figure 10:**
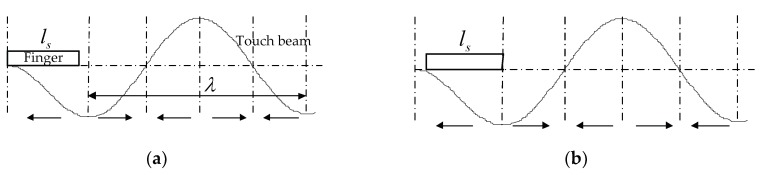

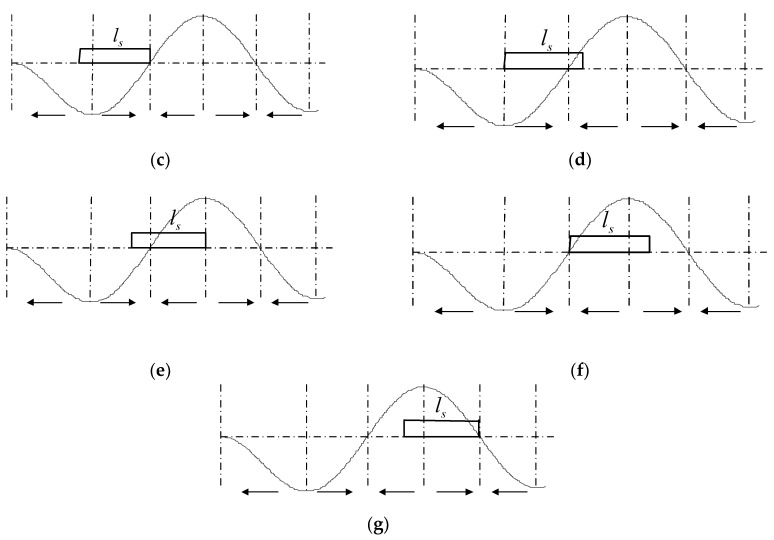
Key positions of the finger on the cantilever beam. (**a**) Position I; (**b**) position II; (**c**) position III; (**d**) position IV; (**e**) position V; (**f**) position VI; (**g**) position VII.

One cantilever touch beam was selected as the research object, and the relevant parameters were the same as in Table 1, Table 2 and Table 3. One operating mode of the touch beam is shown in Figure 10, and the covered length of the finger was considered as *l*_s_ = 0.01 m. In the course of the uniform movement of the finger on the touch beam, according to the theory of the ciliary body’s anisotropic vibration, the ratio of the ciliary bodies of the first whole wave segment, *a*/*u*, exists in six stages. Then, seven key positions of the finger exist on the touch beam. The change rules were as follows:

- As shown in Figure 10, when the finger is moved from the position I to the position II, the covered portion of the finger is all in the opposite directional vibrating region, and the ratio, *a/u*, of the same direction of the vibrating ciliary bodies is maintained at 0.
- When the finger moves from position II to position III, the same directional vibration area of the finger-covering part gradually increases. Also, the ratio of the same-directional ciliary bodies increases and the increased ratio is (*x* − 0.0131)/*l_s_*.
- When the finger moves from position III to position IV, the ratio of the same direction of ciliary bodies, *a/u*, becomes maximum and remains unchanged. The proportion of the same direction of ciliary bodies in this stage is:
  (13) $$\frac{a}{u}=\frac{\lambda }{4l_{s}}.$$
- When the finger moves from position IV to position V, the opposite directional vibrational area of the finger-covering part gradually increases. Additionally, the ratio of the same directional ciliary bodies decreases from *λ*/(4*l_s_*), and the decreased ratio is (*x* − 0.0131 − *λ*/2)/*l_s_*.
- When the finger moves from position V to position VI, the increasing proportion of the same direction of ciliary bodies is equal to the decrease. So, the proportion remains unchanged, and the proportion of the same direction of ciliary bodies in this stage is:
  (14) $$\frac{a}{u}=\left(l_{s}-\frac{\lambda }{4}\right)/l_{s}=1-\frac{\lambda }{4l_{s}}.$$
- When the finger moves from position VI to position VII, the same directional vibration area of the finger-covering part gradually increases. The ratio of the same-directional ciliary bodies increases from 1 − *λ*/(4*l_s_*), and the increased ratio is (*x* − 0.0331 − *λ*/2)/*l_s_*.

After that, all the whole wave segments met the above change rules 3 to 6. So, the coefficient, *γ*, was introduced to indicate the number of the whole wave segments after the first. In summary, according to the change rules of the ratios of the same directional ciliary bodies in the five stages, the relationship between *a*/*u* and the positional coordinates are as follows:

(15) $$\frac{a}{u}=\{\begin{array}{cc} 0 & x\in [0,0.0131)\\ \frac{x-0.0131}{l_{s}} & x\in [0.0131,0.0218)\\ \lambda /4l_{s} & x\in [0.0218+\frac{\lambda \gamma }{2},0.0231+\frac{\lambda \gamma }{2})\\ \frac{\lambda }{4l_{s}}-\frac{x-0.0231-\frac{\lambda \gamma }{2}}{l_{s}} & x\in [0.0231+\frac{\lambda \gamma }{2},0.0318+\frac{\lambda \gamma }{2})\\ 1-\lambda /4l_{s} & x\in [0.0318+\frac{\lambda \gamma }{2},0.0331+\frac{\lambda \gamma }{2})\\ 1-\frac{\lambda }{4l_{s}}+\frac{x-0.0331-\frac{\lambda \gamma }{2}}{l_{s}} & x\in [0.0331+\frac{\lambda \gamma }{2},0.0418+\frac{\lambda \gamma }{2}) \end{array},$$

where *γ* ∈ Z, 0 ≤ *γ* ≤ 5, *x* ≤ 0.1.

**Figure 11:**
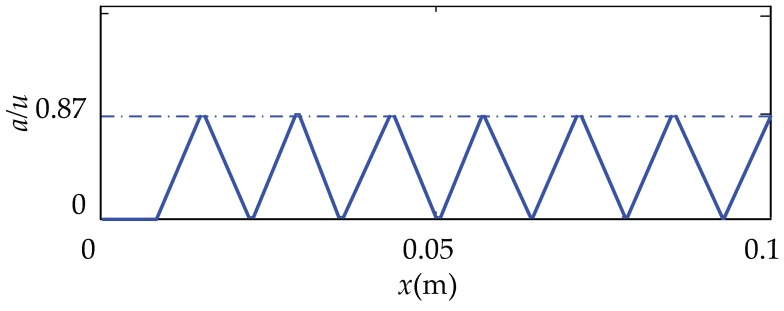
The relationship between the ratio, *a/u*, and the position coordinates, *x*.

From Equation (15), the relationship between the proportion of the same directional ciliary bodies and the positional coordinates is shown in Figure 11. From the image, we know that the ratio, *a/u*, increases from zero and then decreases when the finger moves from left to right, and periodically alternates in the interval of [0,0.87]. The value of *a/u* maintains a shorter distance when it is at a maximum or minimum position.

**Figure 12:**
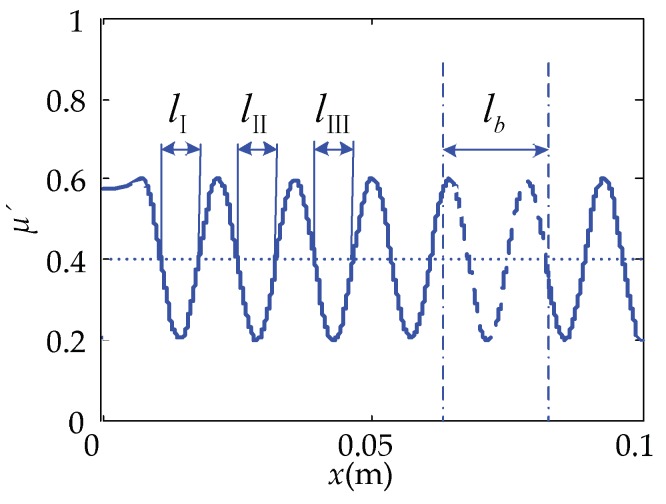
The relationship of *μ*’-*x* at the operating frequency of 24,221 Hz.

Substituting Equation (15) into (12), the relationship between the equivalent friction coefficient and the position of the touch beam at one operating mode of the cantilever touch beam can be obtained, as shown in Figure 12. The length of the dotted line, *l*_b_, is the piezoelectric sheet pasted length. From Figure 12, the following observations were worth noting:

The equivalent friction coefficient of the touch beam also alternates periodically and fluctuates around 0.2 to 0.6 and centered at *μ*’ = 0.4. There are four troughs of the equivalent friction coefficient on the left side of the length, *l_b_*, of the piezoelectric sheet, which is the place where the equivalent friction coefficient is relatively low and is also a relatively smooth position. However, the distance between the fourth valley (from left to right) and the segment where the piezoelectric sheet is located is short, and it is difficult to move in the length of this segment during the actual touch process. Therefore, the first three valleys, *l*_I_, *l*_II_, and *l*_III_, are the three easily perceived smoother positions on the left side of the piezoelectric sheet. Taking the position of the trough at each position as the base point, they are respectively located at 0.0137, 0.0275, and 0.0422 m on the abscissa, *x*.

## 4. Effect of System Parameters on Tactile Changes

### 4.1. Effect of System Parameters on Tactile Changes in Full-Coverage 

**Figure 13:**
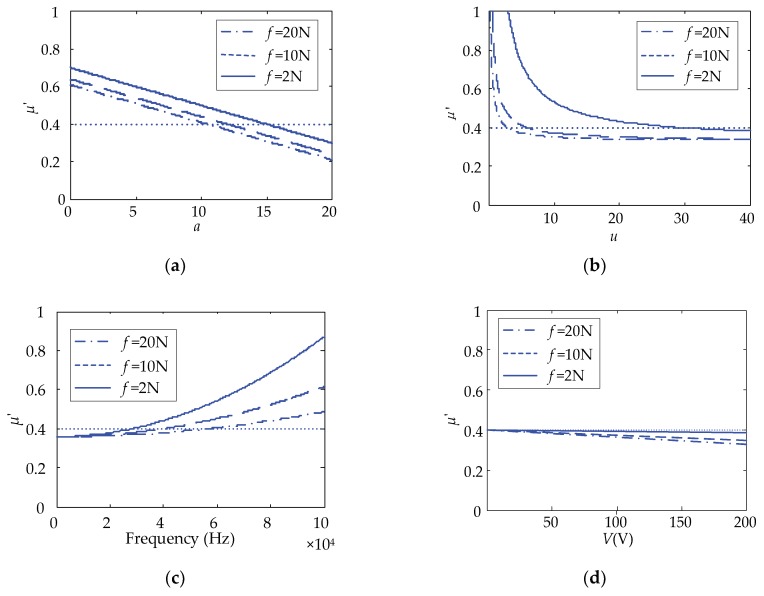
The effect of key parameters on the equivalent friction coefficient in full-coverage. (**a**) *μ’*-*a* relational figure; (**b**) *μ’-u* relational figure; (**c**) *μ’*-frequency relational figure; (**d**) *μ’*-*V* relational figure.

Four parameters were selected to analyze the effect on the equivalent friction coefficient, including the number of the same directional ciliary bodies, *a*; the total number of ciliary bodies, *u*; the operating frequency, *ω*; and the excitation voltage amplitude, *V*. Figure 13 shows the variation of the equivalent friction coefficient with the parameters changed under the conditions of 2, 10, and 20 N finger pressures.

From Figure 13, the following observations were worth noting:

- As *a* increases, the equivalent friction coefficient, *μ’*, of the full-coverage touch beam gradually becomes smaller, and the two are linearly negatively correlated. As *u* increases, the equivalent friction coefficient, *μ’*, of the full-coverage touch beam also gradually becomes smaller.
- However, as *ω* increases, the equivalent friction coefficient, *μ’*, of the full-coverage gradually increases. Additionally, with the increase of *V*, the equivalent friction coefficient, *μ’*, of the full-coverage touch beam gradually decreases linearly. Comparing the *μ’* curves under the three touch pressures, the higher the touch pressure, *f*, is, the smaller the value of *μ’* becomes.
- Under the full-coverage touch, the change of the parameter, *a*, and frequency has the greatest influence on the equivalent friction coefficient. Although *u* has a great influence on the friction coefficient at the beginning, with the increase of *u*, the effect after 20 is not significantly changed. In order to achieve the best results, the same directional ciliary bodies, *a*, and voltage, *V*, should be increased as much as possible, and the parameter *u* can be kept within 20.

### 4.2. Effect of System Parameters on Tactile Changes in Local-Coverage

**Figure 14:**
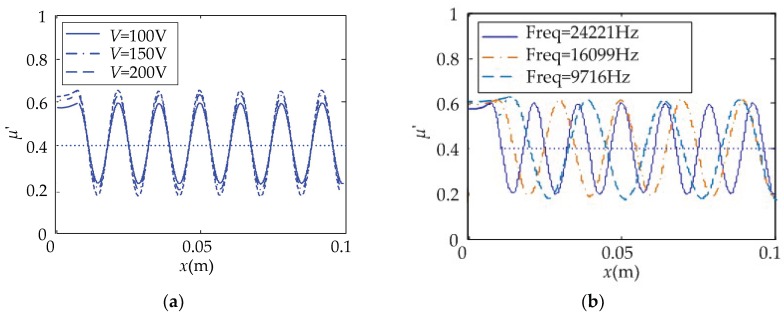
The changes of *μ’-x* under the effect of operating parameters in local-coverage. (**a**) Operating voltage changed; (**b**) operating frequency changed.

The parameters of the operating voltage, *V*, and frequency, *ω*, of the local-coverage cantilever touch beam were changed. The relationship between the equivalent friction coefficient and the position of the touch beam under the effect of the operating parameters was obtained, as shown in Figure 14.

From Figure 14, the following observations were worth noting:

As the operating voltage amplitude, *V*, increases, the *μ’* variation amplitude of the touch beam gradually increases with 0.4 as the center, and the corresponding position becomes smoother or rougher. With the decrease of the operating frequency, *ω*, the *μ*’ variation amplitude of the touch beam slightly increases with 0.4 as the center. However, when the frequency decreases, the number of peaks and troughs gradually decreases.

## 5. Experiments

### 5.1. The Experiments on the Sliding Friction of the Cantilever Touch Beam

**Figure 15:**
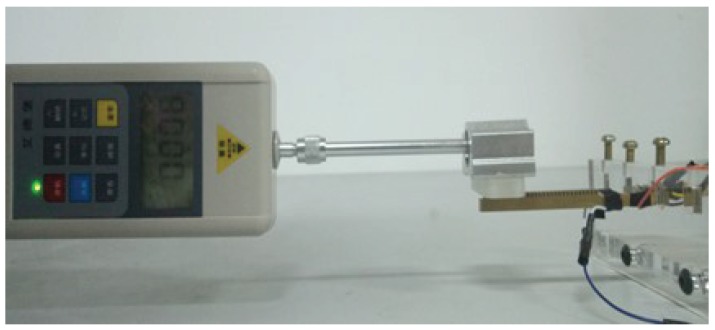
The sliding friction force test.

A cantilever touch beam with a ciliary body spacing of 1 mm was used as the experimental object. One sine signal with a voltage amplitude of 100 V and a frequency of 23,200 Hz was supplied to the touch beam. An acrylic touch block with a weight of 0.56 N was slowly moved on the touch beam from left to right. The real-time friction force change of the block on the touch beam was measured by a digital dynamometer. The test environment is shown in Figure 15.

**Figure 16:**
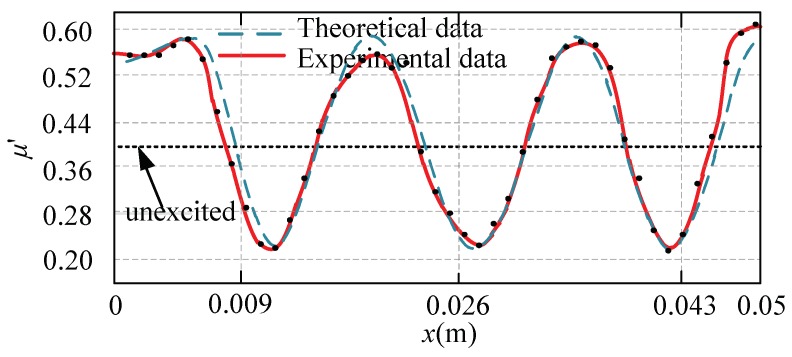
The comparison of the theoretical and experimental data.

From Figure 16, the following observations were worth noting:

By comparing the results of local-coverage tactile theory calculations with the experimental test results, the variation trend of the equivalent friction coefficient obtained from the experiment is very close to that of the theoretical calculation. The test results are very similar to the theoretically calculated positions, except that there is a slight error between the theoretical value and the test value at the first trough, while the value of second trough and third trough are very close to the calculated result. However, the error between the test value and the theoretical value is larger at the two peaks of the equivalent friction coefficient, which we think was caused by the jitter of the thrust of the block when the friction coefficient increases. In general, however, the overall trend of change in the test data shows the validity of the theoretical analysis.

### 5.2. The Experiments of Human Factor 

**Figure 17:**
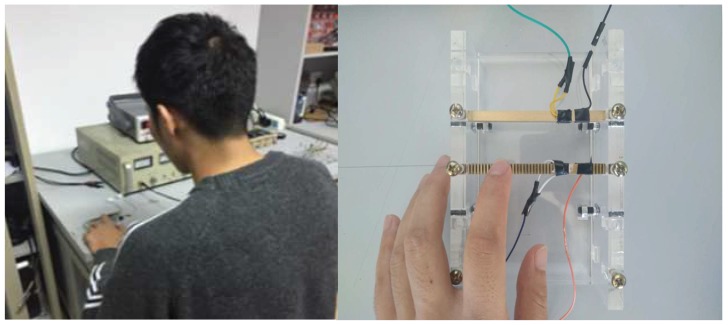
The experiments of human factor.

In order to verify the tactile perception effect of the piezoelectric tactile feedback device, nine of the subjects were invited to perform a touch operation on the tactile device, and the tactile perception effect was evaluated. The subjects were random personnel unrelated to the study. The test environment is shown in Figure 17, and the experimental steps were as follows:

- In the case that no signal was supplied to the touch beam, one subject touched the cantilever touch beam with his left index finger and remembered the current tactile sensation.
- A drive signal with a voltage amplitude of 100 V was supplied to the touch beam. Then, the subject was required to touch the ciliary body touch beam again. By comparing this with the tactile feel of the touch beam when no signal was supplied, the subject was questioned to describe the tactile sensory changes about the roughness.
- The operating parameters of the touch beam and the movement direction of the finger were changed. The subject was required to touch the ciliary body touch beam again. The subject needed to describe the changes in the roughness of the touch beam, and we recorded whether the results were consistent with expectations. If the expectations were met, the subject needed to evaluate the degree of tactile perception and we recorded the score.
- Nine subjects were required to perform tests according to steps 1 to 3 and the relevant experimental scores were recorded.

In order to make the test more accurate and comprehensive, the five parameters of operating frequency, operating voltage peak, beam structure, touch direction, and touch pressure were changed in step 3. The specific test methods for the five parameters were as follows:

- The subjects touched the beam from left to right slowly and the frequency of the operating signal was increased. The subjects were required to describe the changes in tactile sensations after touching. The degree to which the tactile sensations changed as the frequency was close to the resonance point was evaluated, and then scores were recorded.
- The ciliary bodies’ structure was changed. Three kinds of ciliary body space, no ciliary body, 1.5 mm, and 1 mm, were selected. The subjects were asked to touch a smooth position of the beam. The subjects were required to describe the changes in tactile sensations after touching. The degree which the tactile sensations changed as the ciliary bodies’ density increased was evaluated and then the scores were recorded.
- The voltage amplitude of the operating signal was regulated from 0 to 200 V while touching. The subjects were required to describe how they felt after they finished touching it. The degree to which the tactile sensations changed as the voltage increased was evaluated and then the scores were recorded.
- The subjects touched the beam from left to right slowly, and then the movement direction of the finger was reversed to touch the beam from right to left. The subjects were required to describe the tactile change. Whether the smooth and rough positions alternated in different directions of the finger motion was queried, and the perceived degree of subjects was scored and recorded.
- The subjects were asked to touch a smooth position of the beam and increase the touch pressure slightly. The subjects were required to describe their feeling after they finished touching the beam. Whether the subjects felt smooth with increasing pressure was queried, and the perceived degree of subjects was scored and recorded.

The scoring guidelines were as follows:

The score was 0 to 10 points, with 0 being no effect; 1–4 being very weak; 5–7 being general; and 8–10 being very effective.

After the tests, nine subjects felt that the touch beam became smoother after the signal was applied, and the tactile sensation scores of the five important parameters are shown in Table 4.

From Table 4, the following observations were worth noting:

- The given comprehensive scores of the nine subjects ranged from 7 to 10 points. The highest average score was 9.6 points and the lowest average score was 7.2 points. The average value of the comprehensive was 8.31 points. It showed that that the tactile feedback device performs well and the sensation reproduction effect of the tactile feedback device with piezoelectric ciliary body beams is notable.
- Among the five test items, the tactile control effect of changing the voltage amplitude was the best. The tactile perception effect of changing the touch pressure was the weakest. The reason is that the change of touch pressure had little effect on the equivalent friction coefficient in the range of test pressures, while the equivalent friction coefficient was more sensitive to the voltage change in local-coverage.
- By analyzing the lower scores, the third subject’s scores were lower. He gave only 4 points for the effect of changing the touch pressure. In other test items, he gave 7 or 8 points, which is approximately the scores given by others. It indicates that there is a difference in the sensitivity of human skin receptors when the perception of tactile changes faintly.

## 6. Conclusions

The principle of the anisotropic vibration tactile model of the ciliary body touch beam was explored. The equation of the equivalent friction coefficients in full-coverage and local-coverage of the touch beam was established, and the effects of system parameters on the equivalent friction coefficient were analyzed. An experiment on the sliding friction of the touch beam and the experiment of human factors were conducted.

- The full-coverage was mainly affected by the proportion of the same direction of ciliary bodies and the operating frequency. The greater the proportion of the same direction of vibrating ciliary bodies is, the smaller the full-coverage equivalent friction coefficient is. The greater the operating frequency is, the greater the full-coverage equivalent friction coefficient is.
- The local-coverage was mainly affected by the touch position and the amplitude of the operating voltage. The local equivalent friction coefficient at the contact position alternated periodically, and the left side of the piezoelectric sheets, which was 0.0137, 0.0275, and 0.0422 m, respectively, on the abscissa, *x*, is the relatively smooth position that was easily perceived. The larger the amplitude of the excitation voltage is, the more obvious the tactile change on the touch beam is.
- The experimental results of the sliding friction of the touch beam were basically consistent with the corresponding theoretical calculations. In the human factor experiments, the tactile effect of changing the voltage amplitude and increasing the ciliary body density in the prototype was notable. All the results were consistent with the expectations.

## Figures and Tables

**Table 1:** Related parameters of the touch beam.

L [m]	h [mm]	W [mm]	E [GPa]	*ρ* [kg/m^3^]
0.1	5	5	105	8500

**Table 2:** Related parameters of a piezoelectric sheet.

lp [mm]	hp [mm]	Wp [mm]	E [GPa]	*ρ*_p_ [kg/m^3^]	e_31_ [c/m^2^]
10	0.3	5	76.5	7500	2.0

**Table 3:** Related parameters of the ciliary bodies.

u	ω [Hz]	f [N]	V [V]	m [kg]
20	24,221	20	100	0.01

**Table 4:** The result of the human factor behavior effect experimental test scores.

Subjects	Frequency	Ciliary Body Structure	Voltage Amplitude	Movement Direction	Touch Pressure	Comprehensive Score
1	10	8	10	10	5	8.6
2	10	10	10	8	7	9
3	8	8	8	7	4	7
4	10	10	10	10	8	9.6
5	8	8	10	8	5	7.8
6	8	10	10	10	7	9
7	7	10	9	8	7	8.2
8	7	8	10	7	8	8
9	8	8	10	7	5	7.6
average	8.44	8.89	9.67	8.33	6.22	8.31
